# Adipose Stromal Cells Contain Phenotypically Distinct Adipogenic Progenitors Derived from Neural Crest

**DOI:** 10.1371/journal.pone.0084206

**Published:** 2013-12-31

**Authors:** Yoshihiro Sowa, Tetsuya Imura, Toshiaki Numajiri, Kosuke Takeda, Yo Mabuchi, Yumi Matsuzaki, Kenichi Nishino

**Affiliations:** 1 Department of Plastic and Reconstructive Surgery, Kyoto Prefectural University of Medicine, Kyoto, Japan; 2 Department of Pathology and Applied Neurobiology, Kyoto Prefectural University of Medicine, Kyoto, Japan; 3 Department of Basic Pathology, Fukushima Medical University School of Medicine, Fukushima, Japan; 4 Department of Physiology, Keio University School of Medicine, Tokyo, Japan; 5 Department of Biochemistry and Biophysics, Graduate School of Health Care Sciences, Tokyo Medical and Dental University, Tokyo, Japan; 6 Institute of Medical Science, Tokyo Medical University, Tokyo, Japan; Instituto Butantan, Brazil

## Abstract

Recent studies have shown that adipose-derived stromal/stem cells (ASCs) contain phenotypically and functionally heterogeneous subpopulations of cells, but their developmental origin and their relative differentiation potential remain elusive. In the present study, we aimed at investigating how and to what extent the neural crest contributes to ASCs using Cre-loxP-mediated fate mapping. ASCs harvested from subcutaneous fat depots of either adult P0-Cre/or Wnt1-Cre/Floxed-reporter mice contained a few neural crest-derived ASCs (NCDASCs). This subpopulation of cells was successfully expanded *in vitro* under standard culture conditions and their growth rate was comparable to non-neural crest derivatives. Although NCDASCs were positive for several mesenchymal stem cell markers as non-neural crest derivatives, they exhibited a unique bipolar or multipolar morphology with higher expression of markers for both neural crest progenitors (p75NTR, Nestin, and Sox2) and preadipocytes (CD24, CD34, S100, Pref-1, GATA2, and C/EBP-delta). NCDASCs were able to differentiate into adipocytes with high efficiency but their osteogenic and chondrogenic potential was markedly attenuated, indicating their commitment to adipogenesis. *In vivo*, a very small proportion of adipocytes were originated from the neural crest. In addition, p75NTR-positive neural crest-derived cells were identified along the vessels within the subcutaneous adipose tissue, but they were negative for mural and endothelial markers. These results demonstrate that ASCs contain neural crest-derived adipocyte-restricted progenitors whose phenotype is distinct from that of non-neural crest derivatives.

## Introduction

Multipotent mesenchymal stem cells (MSCs) are a population of adult stem cells that can be easily isolated from various tissues and expanded *in vitro*. Many reports on their multipotency and possible clinical applications have raised hopes for and interest in regenerative medicine [1], [2]. Among MSCs from various sources, adipose stromal/stem cells (ASCs) have particularly attracted attention over the years because subcutaneous adipose tissue is accessible, abundant, and replenishable [3], [4].

ASCs are commonly isolated from a low-density mononuclear cell population from humans and other species based on their selective adherence to plastic surfaces, and their MSC characteristics including self-renewal and multipotency are verified under different culture conditions *in vitro* [5]. The term “stem cells” is commonly used for ASCs, but recent studies have revealed functional and phenotypic heterogeneity of ASCs. For example, p75 neurotrophin receptor (p75NTR)-positive ASCs exhibit higher potential to differentiate into adipocytes, osteoblasts and neuronal cells than p75NTR-negative ASCs [6], [7]. CD105 expression identifies a subpopulation of ASCs with high chondrogenic ability [8]. Li et al have also reported that the CD31-/CD34+ subpopulation in human ASCs has great potential for adipogenic differentiation [9].

Moreover, the developmental origin of ASCs still remains unclear. It has been generally assumed that MSCs arise from the mesoderm and reside within perivascular niche, but several recent studies suggest multiple sources [10], [11]. Small subsets of adipocytes and adipocyte progenitors might be derived from bone marrow (BM) via myeloid intermediates [12]. On the other hand, it has been reported that a small subset of mature adipocytes in the head originates from the neural crest (NC) [13]. Although the contribution of NC to ASCs remains to be clarified, Takashima et al. have provided evidence that Sox1+ neuroepithelium gives rise to BM-MSCs in part through a NC intermediate stage during embryonic development [14]. These NC-derived BM-MSCs are likely to be replaced by non-NC derivatives after birth, but some of them may persist even in the adult [15]. Thus ASCs are likely to be comprised of heterogeneous cell populations with multiple developmental origins, but it is unclear how these origins relate to their cellular behavior both *in vivo* and *in vitro*. In the present study, we used Cre-loxP-mediated fate mapping to identify ASCs of NC origin and to investigate their cellular characteristics.

## Materials and Methods

### Animals

Transgenic mice expressing Cre recombinase under control of either the rat P0 promoter (P0-Cre) [16] or the mouse Wnt1 promoter (Wnt1-Cre) [17] were mated with CAG-CAT-EGFP mice to obtain P0-Cre/Floxed-reporter or Wnt1-Cre/Floxed-reporter mice, respectively. P0-Cre transgenic mice were kindly provided by Dr. Marco Giovannini at INSERM through the RIKEN BioResource Center (RBRC01459). CAG-CAT-EGFP mice [18] were kindly provided by Dr. Jun-ichi Miyazaki (Osaka University, Osaka, Japan) and were bred in our animal facility. Mice were housed under a 12 h light/dark cycle in an SPF facility with controlled temperature and humidity, and were allowed access to food and water *ad libitum*. All experimental procedures and protocols for animals conformed to the National Institutes of Health Guide for the Care and Use of Laboratory Animals and were approved by either the Committee for Animal Research of Kyoto Prefectural University of Medicine or the Animal Care and Use Committees of Keio University.

### Isolation and culture of mouse ASCs

ASCs were isolated as previously described [19], from trunk (abdominal and inguinal) subcutaneous adipose depots of male newborn (7 day-old), juvenile (3 week-old), and adult (4–6 month-old) P0-Cre/Floxed-reporter mice. Trunk fat pads of adult Wnt1-Cre/Floxed-reporter mice and cephalic (head and neck) fat pads of P0-Cre/Floxed-reporter mice were also used in some experiments. For isolation of ASCs, adipose depots were minced and digested using 0.075% collagenase type I (Sigma-Aldrich, St. Louis, MO) for 45 min at 37°C. The cell suspension was centrifuged at 1200 *g* for 5 min to separate floating adipocytes from the stromal vascular fraction (SVF). After filtration of the suspension through a 70-µm nylon strainer to remove cellular debris, the SVF cells were resuspended in Dulbecco's modified Eagle's medium (DMEM; Nacalai Tesque, Kyoto, Japan) containing 10% fetal bovine serum, and plated onto plastic culture dishes at a density of 2×10^4^ cells/cm^2^, followed by incubation at 37°C in 5% humidified CO_2_. After 24 h, non-adherent cells were removed by a medium change. Fresh culture medium was added and replaced every 3 days. After 7 days (80 to 90% confluency) the cells were treated with 0.25% trypsin (Gibco, Carlsbad, CA) and diluted 1∶3 per passage for further expansion. ASCs seeded at a density of 1×10^4^ cells/cm^2^ after the second passage were used for all experiments unless otherwise noted.

### Characterization of ASCs from subcutaneous adipose depots of the transgenic mice

Flow cytometry using a FACSCalibur flow cytometer and CellQuest software (BD Biosciences, San Diego, CA) was performed to characterize ASCs using the following primary antibodies (applied in optimal amounts): PE-conjugated hamster anti-CD29 antibody (Biolegend, San Diego, CA), PE-Cy5-conjugated mouse anti-CD44 antibody (BD Biosciences), APC-conjugated rat anti- Sca-1 antibody (BD Biosciences), APC-conjugated rat anti- CD90 antibody (BD biosciences), and PE-conjugated rat anti-CD105 antibody (eBioscience, San Diego, CA) as mouse MSC markers, PE-Cy5-conjugated mouse anti-CD31 antibody (BD biosciences) as an endothelial marker, PE-Cy5-conjugated mouse anti-CD45 antibody (eBioscience) as a hematopoietic cell marker, and PE-conjugated mouse anti-CD24 antibody (eBioscience) and APC-conjugated mouse anti-CD34 antibody (eBioscience) as preadipocyte markers. Isotype antibody controls for each cell population were used to set the dot-plot intercepts used for the analysis.

### Immunocytochemistry

Immunocytochemistry was performed as previously described [19]. Briefly, cells were fixed with 4% paraformaldehyde (PFA) for 30 min followed by overnight incubation at 4°C with the following primary antibodies: rabbit anti-fibronectin (Calbiochem, La Jolla, CA), mouse anti- glial fibrillary acidic protein (GFAP) (Sigma-Aldrich), mouse anti-Nestin (clone rat-401, Millipore, Bedford, MA), rabbit anti-S100 (DAKO, Glostrup, Denmark), rabbit anti-p75NTR (Abcam, Cambridgeshire, UK), rabbit anti-perilipin (Cell Signaling Technology, Beverly, MA). The cells were then incubated with Alexa Fluor-tagged secondary antibodies either Alexa 488 or Alexa 564 (Invitrogen, Carlsbad, CA, USA). Nuclei were counterstained with 4′,6′-diamidino-2-phenylindole dihydrochloride (DAPI). The cells were observed and photographed under a fluorescent microscope (Nikon E1000 Eclipse, Tokyo, Japan). The number of immunoreactive cells per total cells was counted in nine randomly-selected microscopic fields at x400 magnification using a fluorescence microscope coupled with a CCD-camera (Hamamatsu Photonics, Hamamatsu, Japan) and the mean percentage of p75NTR, S100, and Nestin positive-cells was calculated. Typically up to 500 cells (a minimum of three coverslips per condition) were counted per quantified marker.

### Sorting and culture of GFP+/− ASCs

GFP+ and GFP− ASCs were separated using fluorescence-activated cell sorting (FACS). In brief, ASCs were isolated from the trunk fat pads of adult P0-Cre/Floxed-reporter mice as described above, and second passage ASCs were sorted into GFP+ and GFP− cells by FACS (FACS Aria, BD Biosciences) based on cellular expression of GFP, and cultured separately in 24-well culture plates. The efficiency of FACS-based purification was confirmed under the fluorescence microscope (>90%). The total cell number was also measured with the flow cytometer (FACS Calibur) to obtain a growth curve for each preparation of ASCs.

### Multi-lineage differentiation of sorted GFP+ and GFP- cells

The multi-lineage differentiation potential of each preparation of ASCs was assessed using the Mouse Mesenchymal Stem Cell Functional Identification Kit (R&D Systems, Minneapolis, MN) according to the manufacturer's protocol. Adipogenic differentiation was assessed by either Oil Red O staining or perilipin immunostaining, and the proportion of perilipin-positive mature adipocytes per total cells was quantified in nine randomly-selected microscopic fields at x400 magnification. In total, more than 500 cells were counted (a minimum of three coverslips per condition). Osteogenic differentiation was assessed by either Alizarin red staining or osteopontin immunostaining. For quantification, the area stained with osteopontin was measured using NIH-image software and expressed as a percentage of the total area [20]. Chondrogenic differentiation was induced by pelleting the cells in a 1.5 ml centrifuge tube and culturing them in DMEM/F-12 with chondrogenic supplement containing dexamethasone, ascorbatephosphate, proline, pyruvate, and TGF-*beta*3. Pelleted cells were cultured for 14 days and cryosectioned followed by Alcian blue staining to visualize acid mucopolysaccharides.

### Real-time quantitative polymerase chain reaction (RT-qPCR)

Total RNA was isolated using the Qiagen RNeasy mini-kit (Qiagen, Germany) followed by Superscript VILO® cDNA synthesis kit (Invitrogen). RT-qPCR using the Sybr Green I reagent was performed on the StepOnePlus (Applied Biosystems, Foster City, CA). The oligonucleotide primers for Sox2 (Mm00488369_s1), Oct3/4 (Mm03053917_g1), and Nanog (Mm02019550_s1) were purchased from Applied Biosystems. Other primers used are shown in Table 1. All measurements were performed in duplicate. Expression levels were normalized using the expression of the housekeeping gene GAPDH. The relative amount of transcript was estimated by the standard curve method.

**Table 1:** RT-PCR primer sequences.

Gene	Primer sequences	Accession No.	Product size (bp)
Aggrecan	Forward: 5′-CTCTGACATTTGAGGAGGCA-3′ Reverse: 5′-GGGTATCTGACGGTCTGGTC-3′	NM_007424	142
aP2	Forward: 5′-TTGGTCACCATCCGGTCAGA-3′ Reverse: 5′-TTCCACCACCAGCTTGTCAC-3′	NM_024406	207
C/EBP*δ*	Forward: 5′-CGACTTCAGCGCCTACATTGA-3′ Reverse: 5′-CTAGCGACAGACCCCACAC-3′	NM_007679	171
Col2a1	Forward: 5′-AAACCCCCGAACCCTGAAAC-3′ Reverse: 5′-GTGAGGGGAGGACGGTTGG-3′	NM_031163	133
GATA2	Forward: 5′-AACGCCTGTGGCCTCTACTA-3′ Reverse: 5′-GCTCTTCTTGGATTTGCTGG-3′	NM_008090	111
GAPDH	Forward: 5′-TGCACCACCAACTGCTTAGC-3′ Reverse: 5′-TGGATGCAGGGATGATGTTCT-3′	NM_008084	178
PPAR*γ*	Forward: 5′-GTGCCAGTTTCGATCCGTAGA-3′ Reverse: 5′-GGCCAGCATCGTGTAGATGA-3′	NM_011146	142
Pref-1	Forward: 5′-GACCTGGAGAAAGGCCAGTA-3′ Reverse: 5′-AGGGAGAACCATTGATCACG-3′	NM_010052	100
Sox9	Forward: 5′-CAGCAAGACTCTGGGCAAG-3′ Reverse: 5′-TCCACGAAGGGTCTCTTCTC-3′	NM_011448	63

### Immunohistochemistry

Subcutaneous adipose tissue was excised from trunk and cephalic regions of adult transgenic mice. The tissues were fixed with 4% PFA, cryoprotected, and frozen sections (10 µm thickness) were prepared using a cryostat. Fluorescent immunohistochemistry was performed using the following primary antibodies; rabbit anti-αSMA (Abcam), rat anti-CD31 (BD Biosciences), rat-anti platelet-derived growth factor receptor beta (PDGFRβ: CD140β) (eBioscience), mouse anti-GFAP, rabbit anti-S100, rabbit anti-p75NTR, and rabbit-anti-perilipin. Alexa Fluor-tagged secondary antibodies (Invitrogen) were used for visualization and nuclei were conterstained with DAPI. Stained sections were examined and photographed using scanning confocal laser microscopy (FV1000; Olympus).

### Statistical analysis

Either Student's t-test or one-way ANOVA with Tukey multiple comparison test was used to compare differences between groups. All analyses were conducted with GraphPad Prism 5.0 software (GraphPad, La Jolla, CA).

## Results

### Adipose stromal/stem cells in culture contain neural crest-derived cells

**Figure 1:**
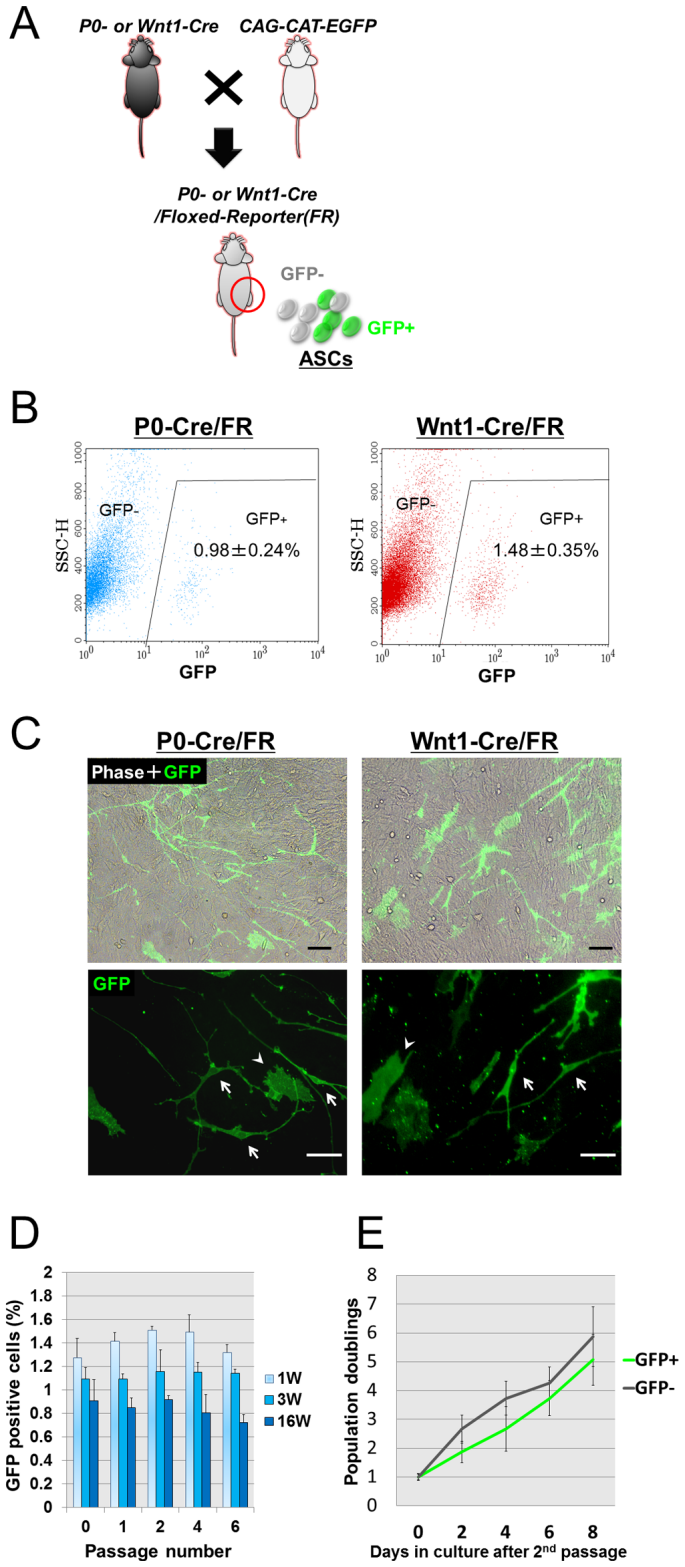
Contribution of neural crest-derived cells to adult ASCs. ***A***. Schematic diagram of the Cre-loxP-mediated fate mapping of NC-derived cells. Either P0-Cre or Wnt1-Cre transgenic mice are crossed with CAG-CAT-EGFP mice to generate NC-specific Cre/Foxed-Reporter (FR) mice in which NC-derived cells can be traced by the expression of the reporter protein GFP. ***B***. Flow cytometry profiles of ASCs from adult Cre/FR mice. The x axis is GFP fluorescent intensity, and the y axis is the side scatter channel (SSC). ASCs from non-transgenic controls served as a gating control. The proportions of GFP+ cells per total cells were 0.98±0.24% and 1.48±0.35% in P0-Cre/FR and in Wnt1-Cre/FR mice, respectively. ***C***. Phase contrast and fluorescent micrographs of second passage ASC cultures isolated from adult P0-Cre/FR (*left*) and Wnt1-Cre/FR mice (*right*). The majority of GFP+ cells possess small soma with long slender processes (*arrow in bottom*), whereas a few cells show a flat/polygonal shape similar to GFP- ASCs (*arrowhead*). Scale bar  = 50 µm. ***D***. The proportions of GFP+ cells in ASC cultures from P0-Cre/FR mice of different ages (1 week-, 3 week-, and 16 week-old) at different passages (from passage 0 to 6). Data are shown as the mean + SEM of 6 independent cultures. ***E***. Growth curves of GFP+ and GFP− cells sorted from second passage ASCs of P0-Cre/FR mice. Results are shown as relative changes to the cell number on day 0 (after sorting). There was no significant difference in the population doubling time between GFP+ and GFP− cells. Data are shown as the mean ± SEM of 3 independent experiments for each condition.

To examine the contribution of NC-derived cells to adult ASCs (NC-derived ASCs; NCDASCs), we performed Cre-loxP-mediated fate mapping using two NC-specific transgenic (Tg) mouse lines; P0-Cre/Floxed-reporter and Wnt1-Cre/Floxed-reporter mice. In these Tg mouse strains, the transient activation of the P0 or Wnt1 promoters in NC cells induces Cre-mediated recombination, which results in NC-derived cells expressing GFP (*Fig. 1A*).

With our isolation and culture methods, we identified a small number of GFP+ cells existing in the second passage ASC cultures isolated from trunk fat pads of adult Tg mice (approx. 1% and 1.5% of total cells in P0-Cre/Floxed-reporter and Wnt1-Cre/Floxed-reporter mice, respectively, *Fig. 1B*). In adherent culture, GFP+ cells exhibited a unique bipolar or multipolar morphology that was distinct from that of the GFP− cells. The majority of GFP+ cells possessed small soma with long slender processes although a few cells showed a flat/polygonal shape similar to the GFP− ASCs (*Fig. 1C*). We compared the yields of NCDASCs in cultures of cells isolated from P0-Cre/Floxed-reporter mice of different ages (*Fig. 1D*). ASC cultures from the younger mice contained more GFP+ cells than those from the older mice, but the differences were less than two-fold (1.40% and 1.12% in 1 week-old and 3 week-old mice, respectively). We next analyzed whether the proportion of GFP+ cells changes during the culture periods. GFP+ cells were still present after 6 passages (over 30 days *in vitro*) and the proportion of GFP+ cells per total cells was not significantly changed from passage 0 to 6, indicating that GFP+ cells are not residual contamination destined to die (*Fig. 1D*). GFP+ cells can grow similarly to GFP− cells under standard ASC culture conditions. However, it remains possible that the survival and growth of GFP+ cells is dependent on GFP− cells. We therefore compared the growth kinetics between GFP+ cells and GFP- cells cultured separately after cell sorting. There was no significant difference in the population doubling time, though GFP+ cells showed a slightly slower growth rate (the approx. population doubling times were 3 and 3.5 days for GFP− and GFP+ cells, respectively) (*Fig. 1E*). In addition, we found no GFP+ cell generated from the GFP− cultures after sorting, indicating that Cre-mediated recombination does not occur *in vitro*.

Together, these findings show that ASC cultures contain a few NC-derived cells whose proliferative potential is comparable to non-NC derivatives under standard culture conditions.

### NCDASCs were positive for mesenchymal stem cell markers but exhibited antigenic features distinct from other ASCs

**Figure 2:**
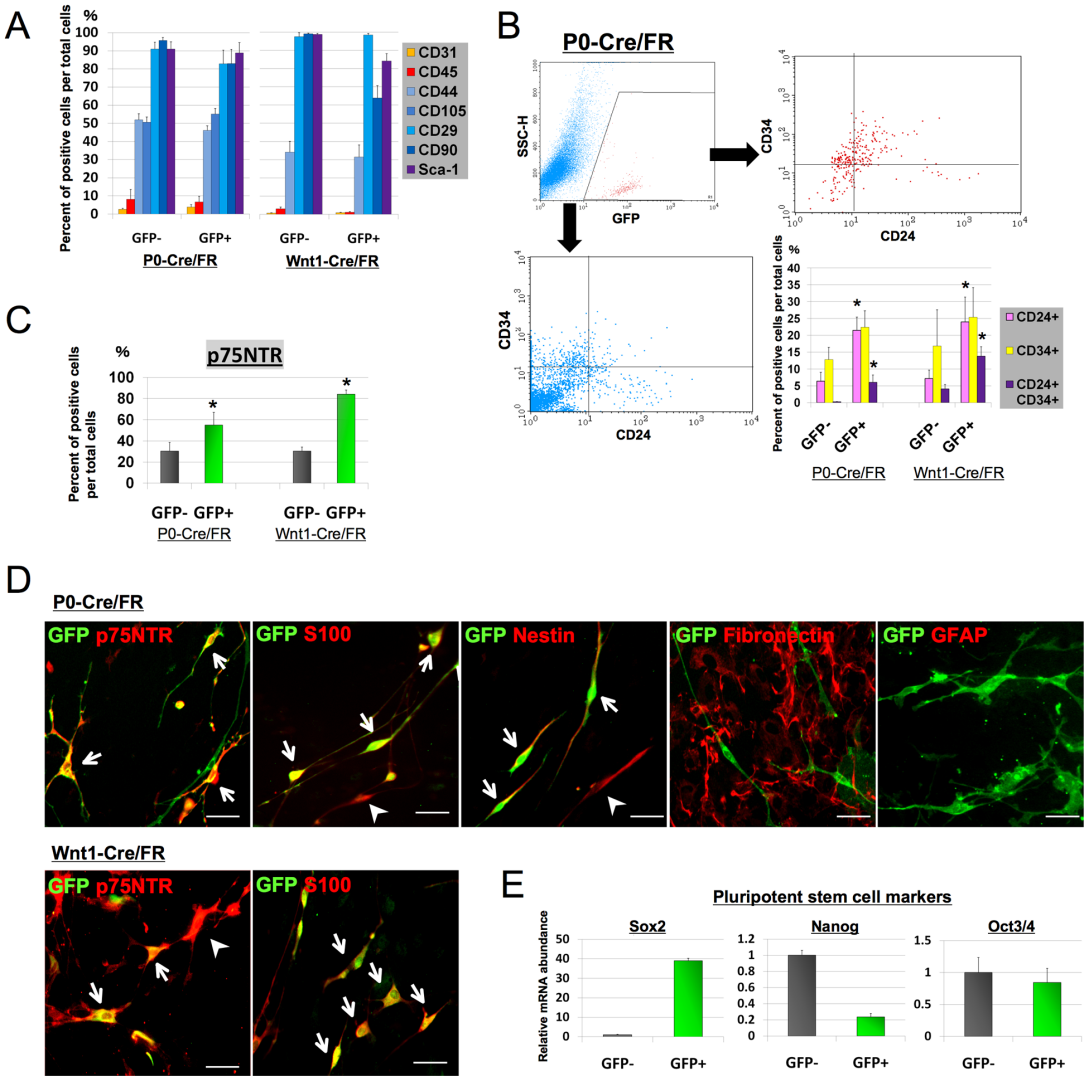
Characterization of neural crest-derived ASCs. ***A***. Quantification of surface marker expression in both GFP+ and GFP− ASCs of either P0-Cre/FR or Wnt1-Cre/FR mice with flow cytometric analysis. The majority of both GFP+ and GFP− cells expressed MSC markers including CD29, CD44, CD90, CD105, and Sca-1. ***B***. The proportion of both CD24- and CD34-positive cells in the GFP+ population was also higher, and the proportion of double-positive cells in the GFP+ population was more than five-fold higher compared to those in GFP− cells. Representative flow cytometry profiles of CD24/CD34 double-positive cells from P0-Cre/FR mice are shown. ***C***. The GFP+ population had a significantly higher proportion of p75NTR-positive cells. Data (*A–C*) are shown as the mean + SEM of 4 independent experiments for each condition. *p<0.05 versus GFP− cells, *t* test. ***D***. Immunofluorescent staining for p75NTR, S100, Nestin, GFAP, and fibronectin (*red*) of either P0-Cre/FR ASCs (*upper five panels*) or Wnt1-Cre/FR (*lower two panels*) shows that the majority of GFP+ cells (*green*) co-expressed p75NTR, S100, and Nestin (*arrow*) but were negative for GFAP and fibronectin. A few GFP-negative but p75NTR- S100-, or Nestin-positive cells were also observed (*arrowhead*). Note that the majority of GFP− cells were positive for fibronectin. Scale bar  = 50 µm. *E*. RT-qPCR analysis for pluripotent markers *Sox2, Nanog*, and *Oct3/4* on FACS-purified GFP− and GFP+ cells from P0-Cre/FR mice. Results are normalized based on *GAPDH* expression and shown as relative changes to GFP− cells. Data are shown as the mean + SEM of 3–4 independent experiments for each condition.

We next characterized the immunophenotype of GFP+ NCDASCs using flow cytometry and immunocytochemistry. Mouse ASCs are known to express several MSC markers including CD29, CD44, Sca-1, CD90, and CD105 [21], [22]. Consistent with our previous report [19], the vast majority of GFP− cells in the second passage ASC culture were positive for these markers. GFP+ cells from adult P0-Cre/Floxed-reporter mice were also positive for all of these markers to approximately the same degree as the GFP− cells. The expression of hematopoietic cell marker CD45 and endothelial cell marker CD31 was minimal. These traditional MSC markers thus failed to distinguish GFP+ cells from GFP− cells (*Fig. 2A*). Then, we further analyzed several other markers; CD34, CD24, and p75NTR. Cells that are positive for both CD34 and CD24 have been reported to represent early adipocyte progenitor cells in adult white adipose tissue [23], [24]. Although CD24/CD34 double-positive cells represented a minor cell population in both the GFP+ and GFP− ASCs, the proportion of the CD24/CD34 double-positive cells was greater than five-fold higher in the GFP+ cells than that in the GFP− cells (*Fig. 2B*). The low affinity neurotrophin receptor p75NTR is expressed in NC stem cells as along with a subpopulation of MSCs [6], [7], [25]. Flow cytometric analysis showed that approximately 60% of total GFP+ cells were positive for p75NTR, which was 2-fold higher than that in the GFP-cells (*Fig. 2C*).

Consistent with the flow cytometry data, immunofluorescent staining showed that 72.4±6.5% of the GFP+ cells coexpressed p75-NTR (*Fig. 2D*). p75NTR+GFP+ cells typically exhibited a bipolar to multipolar morphology. In addition, a greater proportion of GFP+ cells showed both Nestin and S100 immunoreactivity (76.4±17% and 69.3±3.8%, respectively) compared to that of the GFP− cells (2.1%±0.7% and 5.2±1.2%, respectively). Although both p75NTR and S100 can be expressed in Schwann cells, none of the GFP+ cells was positive for GFAP (*Fig. 2D*). It is therefore unlikely that GFP+ cells represent Schwann cell contamination in the ASC culture. GFP+ cells were negative for fibronectin while most GFP-cells were immunoreactive for fibronectin (*Fig. 2D*). We further analyzed the expression of pluripotency markers by RT-qPCR. The expression of *Sox2* was markedly higher (more than 35-fold) but that of *Nanog* and *Oct3/4* was conversely lower in the GFP+ cells relative to the GFP− cells (*Fig. 2E*).

Morphological and immunoreactive characteristics of the GFP+ ASCs cultured from the Wnt1-Cre/Floxed-reporter mice were similar to those from the P0-Cre/Floxed-reporter mice, despite slightly higher proportions of both p75NTR-positive and CD24/CD34 double-positive cells (*Fig. 2A–D*). These results demonstrate that NCDASCs share some MSC markers with other ASCs but display distinct immunophenotypic features.

### Adipogenic differentiation potential of NCDASCs was high but their osteogenic and chondrogenic differentiation was markedly attenuated

**Figure 3:**
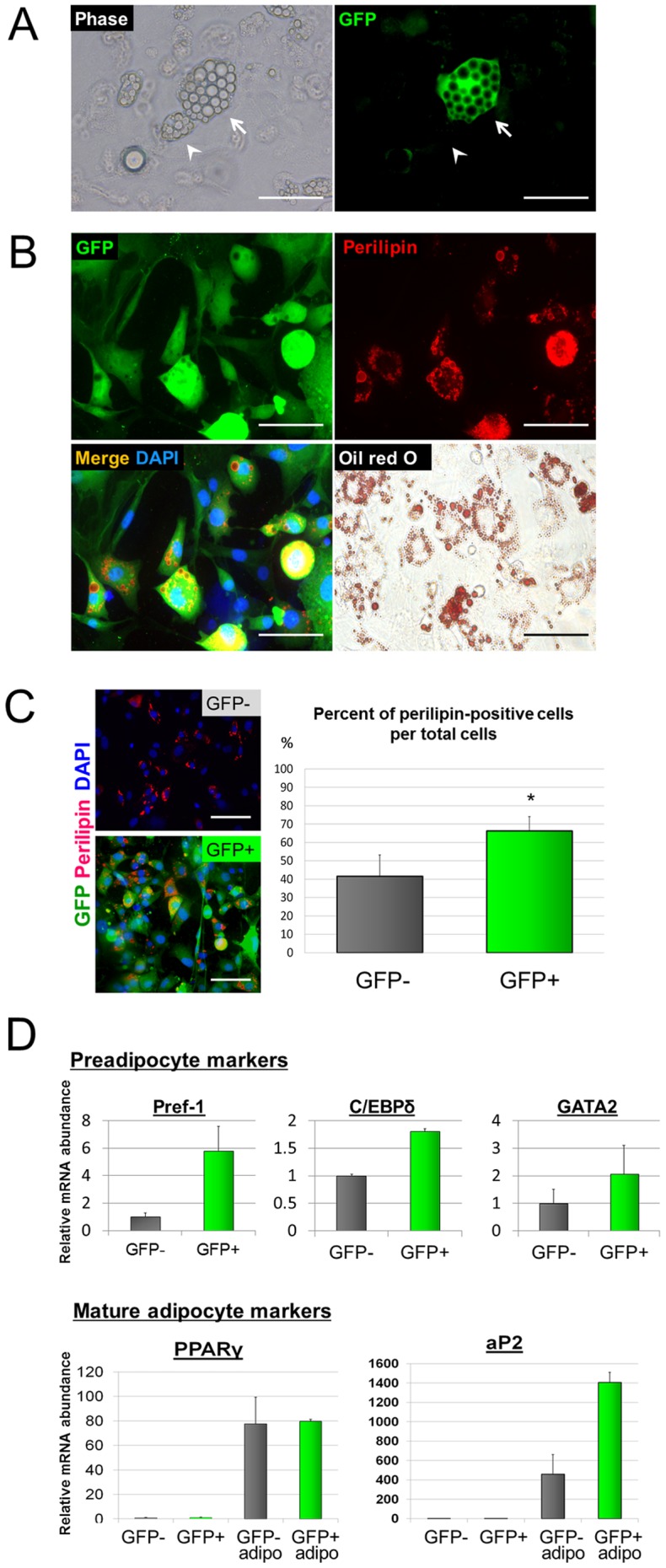
Adipogenic potential of neural crest-derived ASCs. ***A***. Phase contrast and fluorescent images show that both GFP+ (*arrow*) and GFP− ASCs (*arrowhead*) from P0-Cre/FR mice contain lipid droplets after adipogenic differentiation. Scale bar  = 50 µm. ***B***. FACS-purified GFP+ cells are positive for both perilipin immunostaining (*top left, top right, and bottom left*) and Oil red O staining (*bottom right*) after adipogenic differentiation. Nuclei were stained with DAPI (*blue*). Scale bar  = 50 µm. ***C***. Representative images and quantification of adipogenic differentiation of FACS-purified GFP+ and GFP− cells. The percentage of perilipin-positive cells per total cells was more than 1.5-fold higher in the GFP+ cells than that of the GFP-cells. Data are shown as the mean + SEM of 4 independent experiments for each condition. *p<0.05 versus GFP− cells, *t* test. ***D***. RT-qPCR analysis for preadipocyte markers *Pref-1, C/EBPδ*, and *GATA2*, as well as for mature adipocyte markers *PPARγ* and *aP2* on purified GFP− and GFP+ cells before or after (*adipo*) adipogenic induction. Results are normalized based on *GAPDH* expression and shown as relative changes to undifferentiated GFP− cells. Data are shown as the mean + SEM of 3–4 independent experiments for each condition.

We next examined whether the differentiation potential of GFP+ cells is different from that of GFP− cells. To do so, we compared the efficiency of adipogenic, osteogenic, and chondrogenic differentiation between GFP+ and GFP- cells. Five days after being transferred into adipogenic medium, the morphology of both GFP+ and GFP− cells changed from an elongated to a round shape, and lipid vesicle-filled cells were frequently observed 10 days after induction (*Fig. 3A*). We quantitatively analyzed the differentiation potential of GFP+ and GFP− cells which had been sorted and then cultured separately. Although both cell populations effectively differentiated into the adipogenic lineage as assessed by perilipin immunostaining and Oil-red O staining, the ratio of perilipin-positive lipid vesicle-filled cells per total cells was more than 1.5-fold higher in the GFP+ cells when compared with that in the GFP-cells (*Fig. 3B, C*). We also compared the expression of several adipocyte differentiation markers between the GFP− and GFP+ cell populations by RT-qPCR. The expression of preadipocyte/early adipocyte differentiation markers *preadipocyte factor-1 (Pref-1), CCAAT/enhancer-binding protein delta (C/EBPδ)*, and *GATA binding protein 2 (GATA2)* was higher in the GFP+ cells before adipogenic induction relative to the GFP− cells by 580%, 181%, and 206%, respectively (*Fig. 3D*). Although the expression of adipocyte maturation markers *peroxisome proliferator-activated receptor gamma (PPARγ)* and *adipocyte Protein 2 (aP2)* was low in both GFP+ and GFP− cells in an undifferentiated state, the expression of these markers greatly increased after adipogenic differentiation. The expression patterns of these adipogenic genes suggest that both GFP+ and GFP− cells contain adipogenic progenitors but GFP+ cells are more committed into the adipocyte lineage.

**Figure 4:**
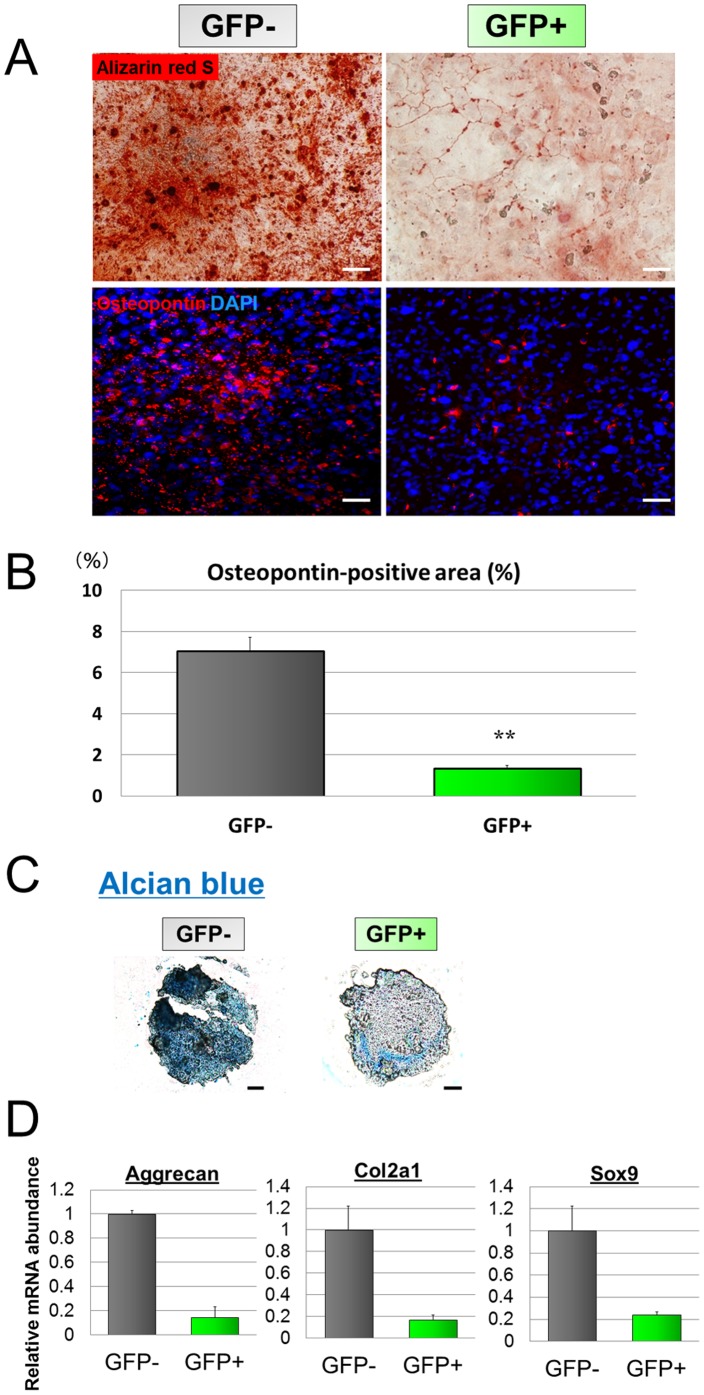
Osteogenic and chondrogenic potential of neural crest-derived ASCs. ***A***. Bright-field images of alizarin staining (*top*) and fluorescent images of osteopontin immunostaning (*red in bottom*) in FACS-purified GFP- and GFP+ cells after osteogenic induction. Nuclei were stained with DAPI (*blue*). Scale bar  = 50 µm. ***B***. The osteopontin-positive area was measured and expressed as a percentage of the total area. Data are shown as mean +SEM, n = 3. **p<0.01 versus GFP− cells, *t* test. ***C***. Bright-field images of Alcian blue staining in FACS-purified GFP+ and GFP− cells after chondrogenic induction. Scale bar = 100 µm. ***D***. RT-qPCR analysis for chondrogenic markers *Aggrecan, COL2a1*, and *Sox9* in the GFP− and the GFP+ cells after chondrogenic differentiation. Results are normalized based on *GAPDH* expression and shown as relative changes to GFP-cells. Data are shown as the mean + SEM of 3–4 independent experiments for each condition.

In contrast to adipogenic potential, considerable mineralization was observed in the GFP-cells but was only faint in the GFP+ cells after osteogenic induction (*Fig. 4A*). Immunoreactivity for the osteogenic differentiation marker osteopontin was similarly greater in the GFP− cells. Quantification of osteopontin-positive area in the culture dish showed a 5.2-fold higher staining in the GFP- cells than that in the GFP+ cells (*Fig. 4B*). We also evaluated the level of chondrogenesis by staining cell pellets with Alcian blue after chondrogenic induction. Diffuse strong staining was observed in the cell pellets generated from the GFP− cultures, whereas the cell pellets from the GFP+ cultures showed much weaker focal staining (*Fig. 4C*). To further verify chondrogenesis, RT-qPCR was performed to compare the expression of chondrogenic markers *Aggrecan*, *collagen type2a1 (Col2a1)*, and *Sox9* [26], [27]. The results showed that the expression of all three markers in the GFP+ cells were markedly lower than that in the GFP- cells after chondrogenic induction (*Fig. 4D*).

These results suggest that NCDASCs are more committed to the adipocyte lineage and their osteogenic/chodrogenic differentiation potential is markedly attenuated compared to that of non-NC-derived ASCs.

### *In vivo* localization of neural crest-derived cells in subcutaneous adipose tissue

**Figure 5:**
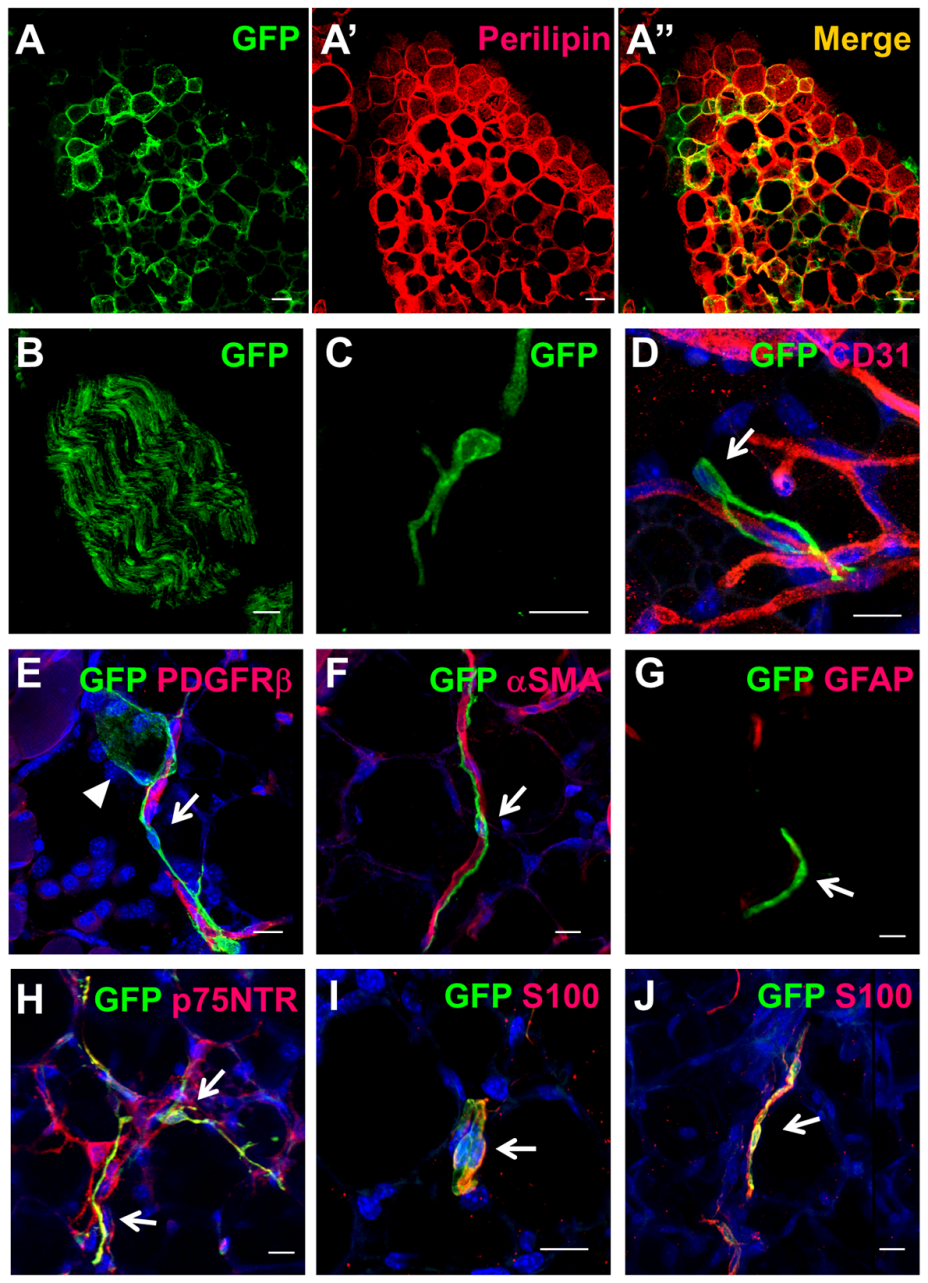
*In vivo* localization of neural crest-derived cells in the subcutaneous adipose tissue. Confocal micrographs of the reporter protein GFP (*green*) combined with immunofluorescence of various markers (*red*) in the adult subcutaneous tissue of either P0-Cre/FR (*A, B, D–I*) or Wnt1-Cre/FR (*C, J*) mice. ***A–A″***. A small portion of perilipin-positive adipocytes expressed GFP in the cephalic region. ***B***. Peripheral nerve tissue was GFP+. ***C***. GFP+ cells with slender processes were identified in the stroma of both cephalic and trunk regions. ***D–J***. These cells (*arrow*) were localized along the vessels or occasionally in the vicinity to GFP+ adipocytes (*arrowhead in E*). They were negative for an endothelial marker CD31 (*D*), mural markers PDGFRβ and αSMA (*E, F*), and a glial marker GFAP (*G*), but positive for p75NTR and S100 (*H–J*). Nuclei were stained with DAPI (*blue, D–F, H–J*). Scale bar = 20 µm (*A, B*), 10 µm (*C–J*).

Since the results so far indicate that adult fat depots contain NC-derived adipogenic progenitors, we next examined whether NC-derived mature adipocytes exist in adult subcutaneous adipose tissue. We found almost no GFP+ adipocyte in the truncal subcutaneous adipose tissue, but a few scattered GFP+ adipocytes were identified in the cephalic region (*Fig. 5A*). In addition to robust GFP expression in peripheral nerves (*Fig. 5B*), small GFP+ cells with slender processes were identified along the vessels in the stroma of both trunk and cephalic subcutaneous tissue (*Fig. 5C*). Despite their perivascular location, these cells were negative for both mural markers (αSMA and PDGFRβ) and an endothelial marker CD31 (*Fig. 5D–F*). These cells were positive for p75NTR and S100 but negative for GFAP (*Fig. 5G–J*). Thus the morphological and immunophenotypic features of these cells are similar to NCDASCs *in vitro*.

Together, these findings show that NC-derived cells reside in the stroma of the subcutaneous fat tissue but the contribution of the NC to *in vivo* adipogenesis in adults is almost none (trunk region) or very low (cephalic region).

## Discussion

In the present study, we analyzed the contribution of NC cells to ASCs using Cre/loxP fate mapping and demonstrate that 1) a small subpopulation of ASCs are derived from the NC; 2) the morphological and antigenic features of NCDASCs are distinct but partly overlapping with non-NC derivatives; 3) the differentiation potential of NCDASCs is largely restricted to adipogenesis.

We used two Cre deleter lines, P0-Cre and Wnt1-Cre, to trace NC-derived cells in ASC culture. The P0 protein is a major component of peripheral nerve myelin, but its promoter is transiently active in migrating NC cells during embryonic development [28]. Wnt1 is expressed in the embryonic dorsal tube at the time of NC formation [17]. Although both lines are shown to effectively target the progenies of the NC [16], [17], [29], it has also been reported that a possible discrepancy between the two Cre lines exists due to differential Cre expression including non-NC cell lineages [25], [30], [31]. Our results show that both Cre lines targeted the cells with the same features at relatively similar frequencies in ASC culture isolated from adult trunk fat depots. In addition, GFP+ cells were already present in the SVF upon their isolation, and the frequency of GFP+ cells did not significantly change during the culture periods. The GFP- cultures did not generate GFP+ cells after cell sorting. We therefore conclude that GFP+ cells in ASC cultures were not the results of ectopic Cre recombination *in vivo* and/or *in vitro* but they were actually derived from the NC.

It has been reported that differential expression of surface markers (e.g. CD105) can distinguish subpopulations of ASCs [8], [9], but we found no significant difference in the expression of general MSC markers between NCDASCs and non NC-derivatives. Nevertheless, NCDASCs displayed unique antigenic features. First, NCDASCs contained a higher proportion of CD24/CD34 double-positive cells, corresponding to an early adipocyte progenitor immunophenotype [23], [24]. Furthermore, NCDASCs were positive for S100. S100 is commonly used as a glial marker, but is also expressed in adipocyte linage cells [32]. Consistent with the notion, the expression of early adipocyte differentiation genes was higher in NCDASCs compared to non-NC derivatives before adipogenic induction. Second, the majority of NCDASCs were positive for NC progenitor markers p75NTR and Nestin. NCDASCs thus show phenotypic characteristics of both MSCs and NC progenitors [14], [15], [25]. Our results, however, show that NC-derived cells accounted for only 10% of the total p75NTR-positive ASCs in our flow cytometric analysis, suggesting that p75NTR-positive ASCs are comprised of both NC and non-NC derivatives. In this context, it is important to note that some of MSC markers can be expressed by NC cells and vice versa [6], [7], [33]. Interestingly, p75NTR-positive human BM stromal cells have been shown to be composed of functionally different subpopulations although their developmental origins remain unknown [34], [35].

NC-derived multipotent stem cells (NCSCs) can be isolated from various types of adult tissues such as skin, gut, the dorsal root ganglia, BM, and whisker pad [25], [36], [37]. It seems reasonable to assume that adipose tissue contains a similar kind of progenitor cell population, but the differentiation potential of NCDASCs into osteocytes and chondrocytes was markedly attenuated. We also failed to differentiate NCDASCs into the neuronal lineage under NCSC culture conditions (*data not shown*). Consistent with our results, Wrage et al. have reported that approximately 2% of ASCs are NC-derived but they do not contribute to neural differentiation of the culture [38]. NCDASCs expressed Sox2 at higher levels, but this finding is likely to indicate their NC progenitor identity rather than their pluripotency because the expression of other pluripotent markers was relatively low. Indeed, the differentiation potential of NC-derived progenitor cells could be different depending on the anatomical site. The cells from the BM gave rise mostly to myofibroblasts, whereas those from the whisker pad generated neurons and myofibroblasts [25]. However, it remains possible that a limited subpopulation of NCDASCs retain multipotency and/or their differentiation potential is suppressed by some signals such as other ASC-derived factors.

ASCs contain phenotypically and functionally distinct subpopulations of precursor cells [3], [39], [40]. However, the relationship between their differentiation potential *in vitro* and their role in adipogenesis *in vivo* are still uncertain. We identified only a few NC-derived GFP+ adipocytes in the head and almost none in the trunk despite their efficient adipogenic potential *in vitro*. This is consistent with *in vivo* fate mapping of either Sox10-Cre or Wnt1-Cre lineage analysis [13], [41]. One possible explanation is that NCDASCs colonize earlier but are largely replaced by non-NC derivatives before *in vivo* adipogenesis occurs. Consistent with the notion, the contribution of NC to either BM-MSCs or adipogenic progenitors in the head mesenchyme sharply declines with age and NC-derived cells account for a very small proportion of total cells in adults [14], [15], [41]. Alternatively, it is possible NCDASCs play a different role or remain dormant under physiological conditions. For example, it has recently been reported that neither endothelial cells nor hematopoietic cells substantially contribute to *in vivo* adipogenesis [24], but both cell types may act as the source of new adipocytes in response to specific stimuli [12], [42].

MSCs are assumed to originate from pericytes *in vivo* in the subcutaneous fat tissue [43]–[46]. We identified p75NTR-positive NC-derived cells along the vessels in the trunk fat tissue, but almost none were positive for pericyte markers. It is possible that *in vivo* characteristics of NCDASCs are also distinct from those of non-NC-derived MSCs although it remains undetermined whether these cells are actually the source of adipogenic ASCs. A lack of GFAP immunoreactivity in NC-derived cells both *in vivo* and *in vitro* suggests that they are not Schwann cells, but there are phenotypic similarities between these cells and non-myelinated Schwann cells (e.g. the expression of p75NTR, S100, and Sox2). In this context, it is interesting to note that Schwann cells behave as stem cells to generate mesenchymal progenies under specific circumstances [47], [48].

## Conclusion

**Figure 6:**
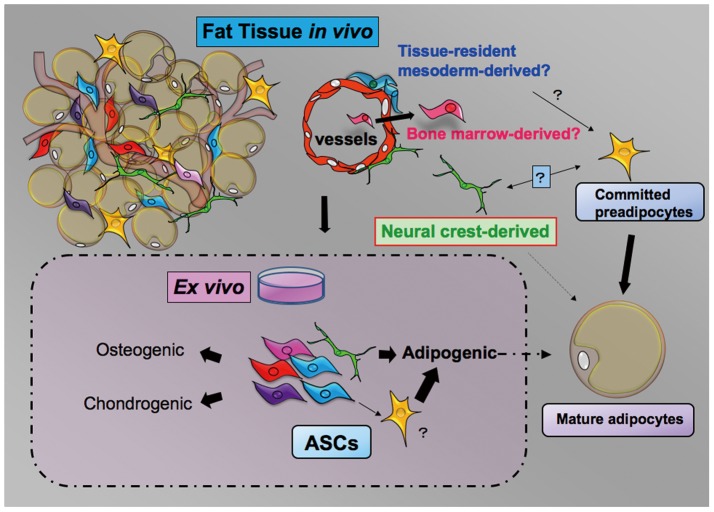
A proposed model showing heterogeneous stem/progenitor cell populations of different origins in adipose tissue. Various kinds of stem/progenitor cells are present in the putative perivascular niche of adipose tissue, such as tissue-resident mesoderm-derived cells, circulating bone marrow-derived cells [12], and NC-derived cells (“Fat tissue *in vivo*”). Endothelial cells could be an alternative source [11], [42]. Although the roles of these different cell populations *in vivo* remain largely unknown, they participate in generating phenotypic and functional diversity of ASCs in culture. NC-derived cells exhibit unique morphological and antigenic features and are largely restricted to the adipogenic lineage. The relationship among multipotent stem cells, osteogenic progenitors, and chondrogenic progenitors and their developmental origins are currently unknown (“*Ex vivo*”). Detailed characterization of different cell subpopulations is important for effective therapeutic application of ASCs.

To summarize, we demonstrated that a minor subpopulation of ASCs is derived from the NC. NCDASCs were successfully expanded under standard ASC culture conditions, but they exhibited several unique features with a relatively adipocyte-restricted differentiation potential. Our results provide evidence that functionally distinct subpopulations of ASCs arise from different developmental origins (*Fig. 6*). Clarification of ASC heterogeneity will be important for effective therapeutic application of ASCs in regenerative medicine.
